# Evaluation of Two Approaches for Aligning Data Obtained from a Motion Capture System and an In-Shoe Pressure Measurement System

**DOI:** 10.3390/s140916994

**Published:** 2014-09-12

**Authors:** Sunwook Kim, Maury A. Nussbaum

**Affiliations:** 1 Industrial and Systems Engineering, Virginia Tech, Blacksburg, VA 24061, USA; E-Mail: sunwook@vt.edu; 2 Virginia Tech–Wake Forest School of Biomedical Engineering and Sciences, Virginia Tech, Blacksburg, VA 24061, USA

**Keywords:** in-shoe pressure measurement, center of pressure, manual material handling

## Abstract

An in-shoe pressure measurement (IPM) system can be used to measure center of pressure (COP) locations, and has fewer restrictions compared to the more conventional approach using a force platform. The insole of an IPM system, however, has its own coordinate system. To use an IPM system along with a motion capture system, there is thus a need to align the coordinate systems of the two measurement systems. To address this need, the current study examined two different approaches—rigid body transformation and nonlinear mapping (*i.e.*, multilayer feed-forward neural network (MFNN))—to express COP measurements from an IPM system in the coordinate system of a motion capture system. Ten participants (five male and five female) completed several simulated manual material handling (MMH) activities, and during these activities the performance of the two approaches was assessed. Results indicated that: (1) performance varied between MMH activity types; and (2) a MFNN performed better than or comparable to the rigid body transformation, depending on the specific input variable sets used. Further, based on the results obtained, it was argued that a nonlinear mapping *vs.* rigid body transformation approach may be more effective to account for shoe deformation during MMH or potentially other types of physical activity.

## 1. Introduction

Tracking center of pressure (COP) locations under the shoe or the foot has broad utility/application in the fields of biomechanics and motor control. COP is the point of application of ground reaction forces (GRFs) that reflect the net force exerted by or on the whole body [1]. In combination with body segmental kinematics and GRFs, COP locations are required to compute joint kinetics using the bottom-up inverse dynamics approach [1,2]. Furthermore, several COP-based measures have been examined in numerous studies [3,4], to assess and understand postural control during, for example, quiet stance and gait, and to assess influences or differences related to age, gender, environmental conditions, health status, *etc.*

A force platform—a conventional system used for COP measurement—is typically fixed in the floor or other structure (e.g., stairs, walkways). Hence, and as highlighted earlier [5,6], the size, placement, and/or number of force platforms can restrict an individual's foot placement and/or the monitoring of multiple footsteps. More generally, a force platform can be difficult to use outside of a laboratory setting. To overcome such limitations, several wearable measurement systems have been introduced, such as instrumented shoe (IS) systems [7–9] and in-shoe pressure measurement (IPM) systems [5,10–12]. IS systems are typically based on two tri-axial load transducers attached externally to the shoe. This allows for measuring loads between the shoe (with the transducer) and the ground, and which can potentially provide data directly comparable to COP measurements obtained from a force platform. Externally attached transducers, however, increase both shoe height and sole rigidity [6,8]. To the authors' knowledge, no study has formally examined how an IS system interacts with different surfaces and terrains (e.g., the effects of increased shoe height and sole rigidity on slip/trip risks). An IPM system uses a pressure-sensitive matrix (*i.e*., insole) that can be fitted into an individual's shoe. The insole captures the plantar pressure distribution below the foot sole, and which can be easily processed to obtain the COP between the foot and the insole. Earlier studies have demonstrated that one commercial IPM system, the Pedar^®^-X, has good measurement accuracy, precision, and repeatability [13,14]. Using an IPM system, *vs.* an IS system, therefore appears to be more practical and valid for use in diverse environments, particularly since the former can be used with an individual's own shoe(s).

The insole of an IPM system has its own coordinate system. As such, using an IPM system along with a motion capture system (such as for assessing segmental loads through inverse dynamics) requires aligning the coordinate systems of both measurement systems. To define a transformation matrix between the two coordinate systems, existing studies have generally assumed a rigid body transformation, and used either direct or indirect methods. The direct method defines a coordinate system for the IPM system by locating insole sensors using a pointer with reflective markers [15], and thus does not require a force platform. The indirect method defines a transformation matrix by fitting COP locations between a force platform and the IPM system, either manually [10] or optimally [16–18]. In the studies noted, normal gait was a common dynamic activity of interest. Interestingly, Chumanov *et al.* [17] reported relatively larger root mean square errors (RMSE) during the starting (0%–10%) and ending stance phases (80%–100%) of gait (RMSE = ∼34–57 mm and ∼7–15 mm in the anterio-posterior (AP) and medio-lateral (ML) directions, respectively), compared to the mid-stance phase (10%–80%) (RMSE = ∼8–10 mm and ∼4–7 mm in the AP and ML directions, respectively). This suggests that the rigid body transformation assumption may be less valid, since a shoe can be bent and/or otherwise deformed to varying degrees during different activities.

In the current work, we examined the performance of an optimally-defined rigid body transformation matrix between the coordinate systems of an IPM and motion capture systems. The activities of interest were those involved in manual material handling (MMH), assuming potential use of an IPM system in the context of occupational ergonomics and safety. Furthermore, and without the assumption of rigid body transformation, four different nonlinear functions were investigated that can map IPM data to COP obtained from a force platform. Specifically, we used a multilayer, feed-forward neural network (MFNN) as a mapping function with each of four different sets of input variables (extracted from IPM system outputs). The performance of the mapping functions was compared to an optimally-defined rigid body transformation.

## 2. Methods

### 2.1. Participants and Experimental Procedures

Ten young participants (19–29 years old, 5 males and 5 females) completed the study. Their mean (SD: range), stature, body mass, and foot length were 171.5 (6.9: 164–183) cm, 76.1 (13.2: 62.5–104.6) kg, and 26.8 (1.4: 24.5–28) cm, respectively. All participants reported being healthy, physically active, and having no current musculoskeletal injuries that limit their normal daily activities. Prior to any data collection, participants completed an informed consent procedure approved by the Virginia Tech Institutional Review Board.

Participants completed each of four simulated MMH activities that were intended to represent a range of occupational demands. A complete description of these activities was presented earlier [19], and as such are only summarized here. All activities were performed with a box (width × length × depth = 33 × 48.3 × 24 cm) with mass set to 20/15% of individual body mass for male/female participants. The specific MMH activities were: (a) symmetric lifting and lowering from ground to individual elbow height, and *vice versa* (LL_GROUND_); (b) symmetric lifting and lowering from a table adjusted to individual knuckle height to individual elbow height and vice versa (LL_KNUCKLE_); (c) asymmetric lifting and lowering from/to tables located at the side of a participant and adjusted to individual knuckle height (LL_ASYM_); and (d) pushing and pulling away from/toward the body (PushPull). The presentation order of activities was randomized, and two trials of each activity were completed. Prior to data collection, participants were allowed to practice each MMH type until they felt comfortable and competent with each. During each trial, participants were asked to keep each foot on a separate force platform, but were allowed to move each foot freely over the force platform surface. Further, participants performed all activities using self-selected comfortable styles and speeds, and were allowed to change their working styles and speeds in each trial as desired.

### 2.2. Instrumentation

External foot kinetics were measured using two force platforms (AMTI OR6-7-1000, Watertown, MA, USA) and a commercial IPM system (Pedar^®^-X, Novel Gmbh, Munich, Germany) with three different insole sizes covering shoe sizes of 24.8–28.3 cm. Each insole contained 99 pressure-sensitive capacitive sensors. Of note, prior to data collection, each insole was calibrated using a trublu^®^ calibration device (Novel Gmbh, Munich, Germany) as recommended by the manufacturer. The force platforms and the IPM system were sampled at 960 Hz and 60 Hz, respectively. Force platform outputs were low-pass filtered (15 Hz cut-off; 2nd order zero-lag Butterworth) and down-sampled to 60 Hz. Foot kinematics were captured at 60 Hz, using a 7-camera optical motion capture system (Vicon MX, Vicon Motion Systems Inc., Denver, CO, USA). Passive reflective markers were placed bilaterally on the calcaneus and 1st and 5th metatarsal heads, and a cluster of three markers was placed over the side of each shoe. Prior to the MMH activities, the 1st and 5th metatarsal head markers were removed after being referenced to the corresponding cluster, and removed markers were reconstructed using the clusters [20]. During data collection, the IPM system was synchronized with other systems via a TTL pulse (Pedar^®^ Sync Box). All off-line data processing was completed using MATLAB 7 (Mathwork™ Inc., Natick, MA, USA). When the vertical GRF from the force platform was <20 N, the foot was considered off the ground and associated COP data were excluded from subsequent analyses described below.

### 2.3. Transformation Matrix, $T_{\text{Insole}}^{\text{Shoe}}$ between the Insole and the Shoe Coordinate System

COP locations from the IPM system (*i.e*., expressed in the insole coordinate system) can be transformed to the global (*i.e.*, motion capture) coordinate system [15] using:

(1) $${\text{COP}}_{i}^{\text{Global}}=T_{\text{Shoe},i}^{\text{Global}}T_{\text{Insole}}^{\text{Shoe}}{\text{COP}}_{i}^{\text{Insole}}$$

where **COP***_i_* is a 3 × 1 position vector at the *i^th^* time frame. $T_{\text{Shoe},i}^{\text{Global}}$ and $T_{\text{Insole}}^{\text{Shoe}}$ are 4 × 4 transformation matrices, respectively from the shoe to the global coordinate system and from the insole to the shoe coordinate system. Using reflective markers placed on the shoe, $T_{\text{Shoe},i}^{\text{Global}}$ can be defined as:

(2) $$T_{\text{Shoe},i}^{\text{Global}}=\left[\begin{array}{c} \begin{array}{cc} \begin{array}{cc} X_{i} & Y_{i} \end{array} & \begin{array}{cc} Z_{i} & O_{i} \end{array} \end{array}\\ \begin{array}{cc} \begin{array}{cc} 0 & 0 \end{array} & \begin{array}{cc} 0 & 1 \end{array} \end{array} \end{array}\right]$$

with

$$\begin{array}{c} Y_{i}=\frac{0.5\left(\text{MT}1_{i}+\text{MT}5_{i}\right)-{\text{HEEL}}_{i}}{\left\|0.5\left(\text{MT}1_{i}+\text{MT}5_{i}\right)-{\text{HEEL}}_{i}\right\|}\\ Z_{i}=\frac{\left(\text{MT}5_{i}-{\text{HEEL}}_{i})\times (\text{MT}1_{i}-{\text{HEEL}}_{i}\right)}{\left\|\left(\mathrm{MT}5_{i}-{\text{HEEL}}_{i})\times (\mathrm{MT}1_{i}-{\text{HEEL}}_{i}\right)\right\|}\\ X_{i}=Y_{i}\times Z_{i} \end{array}$$

where **O***_i_* is a 3 × 1 origin vector for the shoe coordinate system and set equal to **HEEL***_i_* (Z coordinate is zero since COP is planar data). **MT1***_i_*, **MT5***_i_* and **HEEL***_i_* are 3 × 1 position vectors of the 1st and 5th metatarsals, and the heel, respectively.

**Figure 1.**
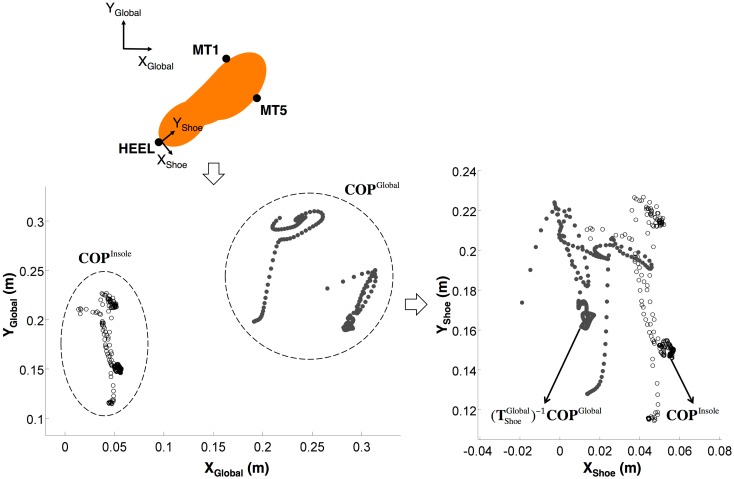
Overview of current transformation problem, specifically involving defining a transformation matrix, $T_{\text{Insole}}^{\text{Shoe}}$ between ${\left(T_{\text{shoe}}^{\text{Global}}\right)}^{-1}$ **COP**^Global^ and **COP**^Insole^. Here, MT1, MT5, and HEEL, respectively, indicate passive reflective markers on the 1st and 5th metatarsal heads and calcaneus. The subscripts Shoe/Global on the axis labels indicate the shoe/global coordinate system.

Pre-multiplying ${\left(T_{\text{shoe},i}^{\text{Global}}\right)}^{-1}$ in Equation (1) enables defining a transformation matrix, $T_{\text{Insole}}^{\text{Shoe}}$ between ${\left(T_{\text{shoe},i}^{\text{Global}}\right)}^{-1}{\text{COP}}_{i}^{\text{Global}}$ and ${\text{COP}}_{i}^{\text{Insole}}$ Figure 1), and $T_{\text{Insole}}^{\text{Shoe}}$ can be solved in two different ways. In the first approach, and similar to existing studies [16–18], $T_{\text{Insole}}^{\text{Shoe}}$ can be assumed as a rigid-body homogeneous transformation matrix:

(3) $$T_{\text{Insole}}^{\text{Shoe}}=\left[\begin{array}{cc} R_{\text{Insole}}^{\text{Shoe}} & d_{o}\\ \begin{array}{ccc} 0 & 0 & 0 \end{array} & 1 \end{array}\right]$$

where $R_{\text{Insole}}^{\text{Shoe}}$ is a 3 × 3 rotation matrix and **d***_o_* is a 3 × 1 translation vector. As in the aforementioned studies, the parameters of the rotation matrix and translation vector can be determined using a numerical search algorithm. Here, instead of a numerical search, we simply considered ${\left(T_{\text{shoe},i}^{\text{Global}}\right)}^{-1}{\text{COP}}_{i}^{\text{Global}}$ and ${\text{COP}}_{i}^{\text{Insole}}$ as corresponding clusters of points in different reference frames, and applied a procedure to determine those rigid body transformation parameters in a least-squares sense, using a singular value decomposition [20]. This transformation method is hereafter referred to as RIGID.

In the second approach, and without assuming a rigid body transformation, we can consider $T_{\text{Insole}}^{\text{Shoe}}$

as a function that maps insole pressure measures to ${\left(T_{\text{shoe},i}^{\text{Global}}\right)}^{-1}{\text{COP}}_{i}^{\text{Global}}$ . Given that a specific functional relationship is unknown, we used a generalized mapping approach, specifically a multilayer, feed-forward neural network (MFNN) with one hidden layer and sigmoid transfer function (see [21] for more details). As there are numerous potential ways to define input variables to a MFNN from pressure insole measures, we considered four, simple sets of input variables that could help in selecting input variables for future work (note that each is obtained separately for the two feet):

- **Principal components (PCs) of insole pressure measures,** ${\text{COP}}_{i}^{\text{Insole}}$ : 99 pressure measures from the insole are converted to forces, and are then divided by half of a participant's body mass. The dimension of these forces is *n* × 99, where *n* is the length of each COP time series. To reduce the column dimension, principal component analysis (PCA)—a well-established statistical dimension reduction technique [22]—is performed, and the first PCs that cumulatively explained >90% of variance are selected. The dimension of **COP**^Insole^ is *n* × 2, excluding Z coordinate data. The MFNN with this set of input variables is referred to as MFNN1.
- **Total force, Ratio of active pressure sensors,** ${\text{COP}}_{i}^{\text{Insole}}$ : The total force (*n* × 1 vector) from the pressure insole is divided by half of a participant's body mass. The ratio of active pressure sensors is computed by dividing the number of active pressure sensors (*i.e.*, pressure value > 0) in each time frame by the total number of pressure sensors (*i.e.*, 99). The MFNN with this set of input variables is referred to as MFNN2.
- **Total force, Ratios of active pressure sensors in eight insole regions,** ${\text{COP}}_{i}^{\text{Insole}}$ : To capture the regional distribution of pressures on the insole, along with the total force described above, the insole is divided into eight regions based on work by Savelberg and de Lange [5]: the lateral and medial parts of the heel, two areas in the mid-foot, three areas in the forefoot, and one area for the toes. The ratio of active pressure sensors in each region is obtained (*n* × 8), so that the total dimension of input variables becomes *n* × 11. The MFNN with this set of input variables is referred to as MFNN3.
- **Force** and **Ratios of active pressure sensors in eight insole regions,** ${\text{COP}}_{i}^{\text{Insole}}$ : Both forces and ratios are obtained from the eight noted foot regions (*i.e., n* × 16), so that the total dimension of input variables becomes *n* × 18. The MFNN with this set of input variables is referred to as MFNN4.

### 2.4. Training and Validation of $T_{\text{Insole}}^{\text{Shoe}}$

For each of the five transformation methods (RIGID, and MFNN1-4), $T_{\text{Insole}}^{\text{Shoe}}$ was trained and tested using two-fold cross-validation at the participant level. As such, the training and the testing were performed twice, using one replication of simple lifting activities (LL_GROUND_ and LL_KNUCKLE_), and with the other replication of all MMH tasks for testing. Of note, the simple lifting activities were used for training, since LL_GROUND_ and LL_KNUCKLE_ can respectively represent dynamic and more static activities, and can be easily performed. For the MFNNs, hidden layer sizes from 3 to 20 were considered during the training, and the final size was selected based on minimizing root mean square (RMS) errors. The final size selected varied from 4 to 20, depending on participants and foot side. To examine the method validity, three comparative measures were obtained for each MMH task trial, separately in the AP and the ML directions, and in the shoe coordinate system: (1) RMS error (RMSE), where errors were the difference between **COP***_i_* estimated using $T_{\text{Insole}}^{\text{Shoe}}$ defined by a given method and ${\left(T_{\text{shoe},i}^{\text{Global}}\right)}^{-1}{\text{COP}}_{i}^{\text{Global}}$ ; (2) peak absolute error (PAE); and (3) the coefficient of determination (r^2^).

### 2.5. Statistical Analyses

Separate three-way, repeated measures of analyses of variance (ANOVAs) were performed on the comparative measures, to examine if performance in mapping **COP**^Insole^ to ${\left(T_{\text{shoe},i}^{\text{Global}}\right)}^{-1}{\text{COP}}_{i}^{\text{Global}}$ was affected by the four MMH task types (*Task*), foot side (*Foot; i.e.*, left *vs.* right), and the specific method used for $T_{\text{Insole}}^{\text{Shoe}}$ (*Method*). All comparative measures were log-transformed prior to statistical analyses to achieve normally distributed residuals. Significant effects were examined further using Tukey's HSD *post-hoc* tests. All statistical analyses were complete using JMP^®^ Pro 11.0 (SAS Institute Inc., Cary, NC, USA) with statistical significance determined when *p* < 0.05. All summary data are presented as means (95% Confidence Intervals).

## 3. Results

**Figure 2.**
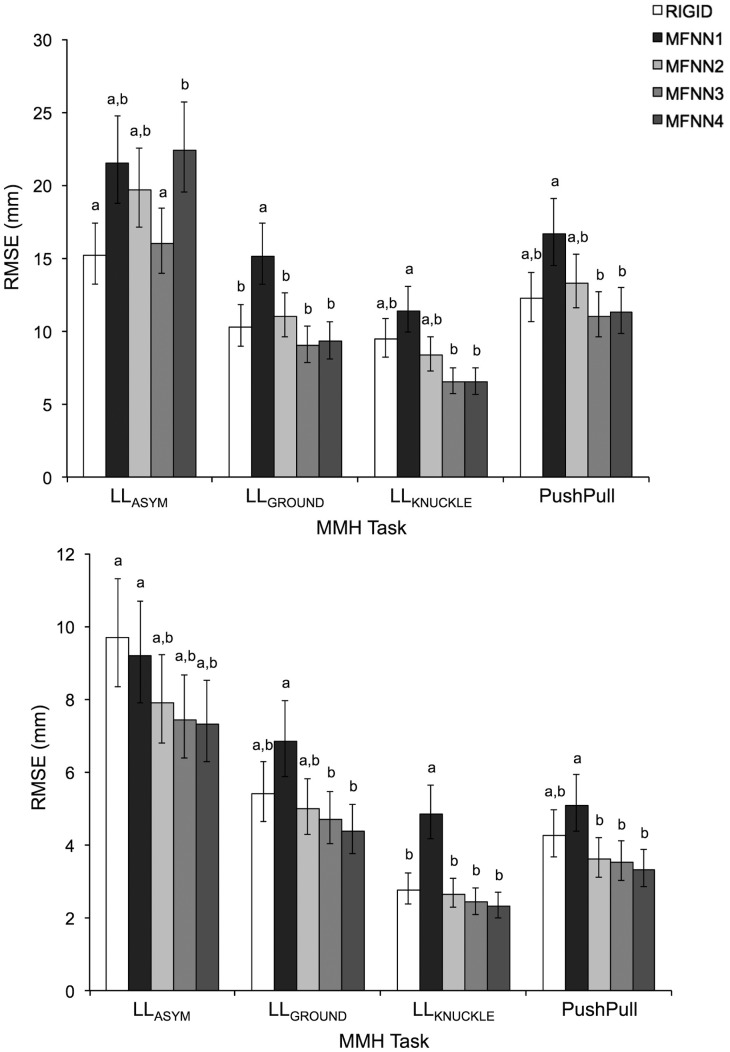
Root mean square errors (RMSE) in the anterio-posterior (AP: **top**) and the medio-lateral (ML: **bottom**) directions for each MMH task type, with respect to the different methods used for $T_{\text{Insole}}^{\text{Shoe}}$ . Note, see the methods section for detailed descriptions regarding the RIGID and MFNN1–4 methods. Pairs of values with different letters are significantly different within a given MMH task type, and error bars indicate 95% confidence intervals.

**Figure 3.**
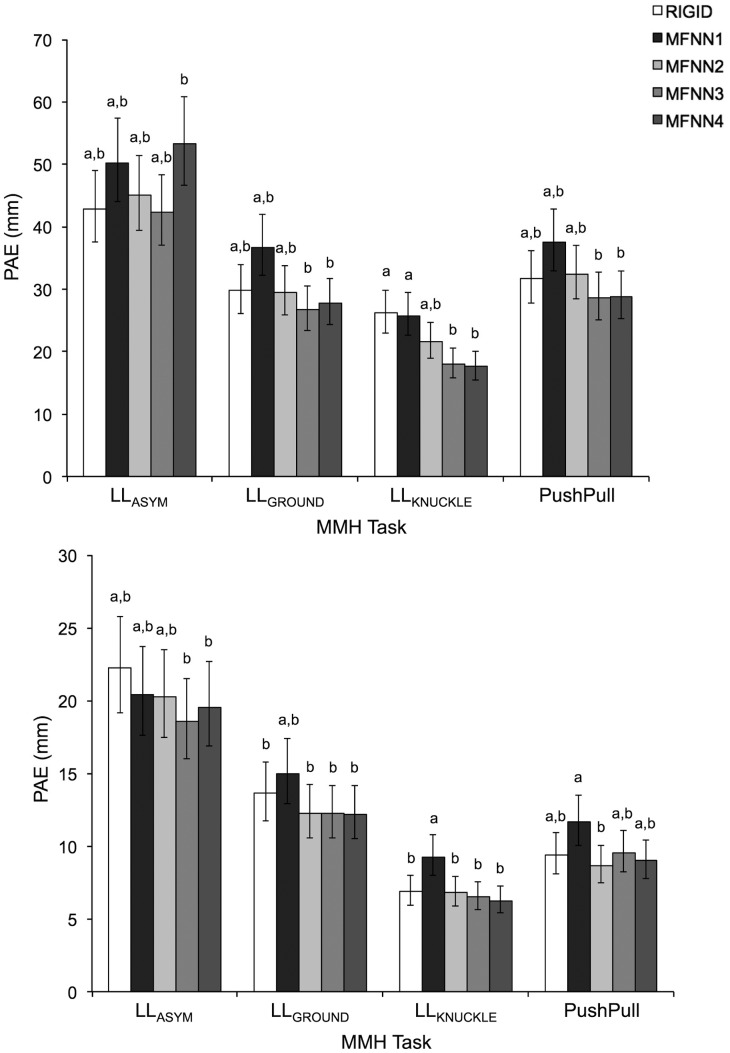
Peak absolute errors (PAE) in the AP (**Top**) and the ML (**Bottom**) directions for each MMH task type, with respect to the different methods for $T_{\text{Insole}}^{\text{Shoe}}$ . Pairs of values with different letters are significantly different within a given MMH task type, and error bars indicate 95% confidence intervals.

**Figure 4.**
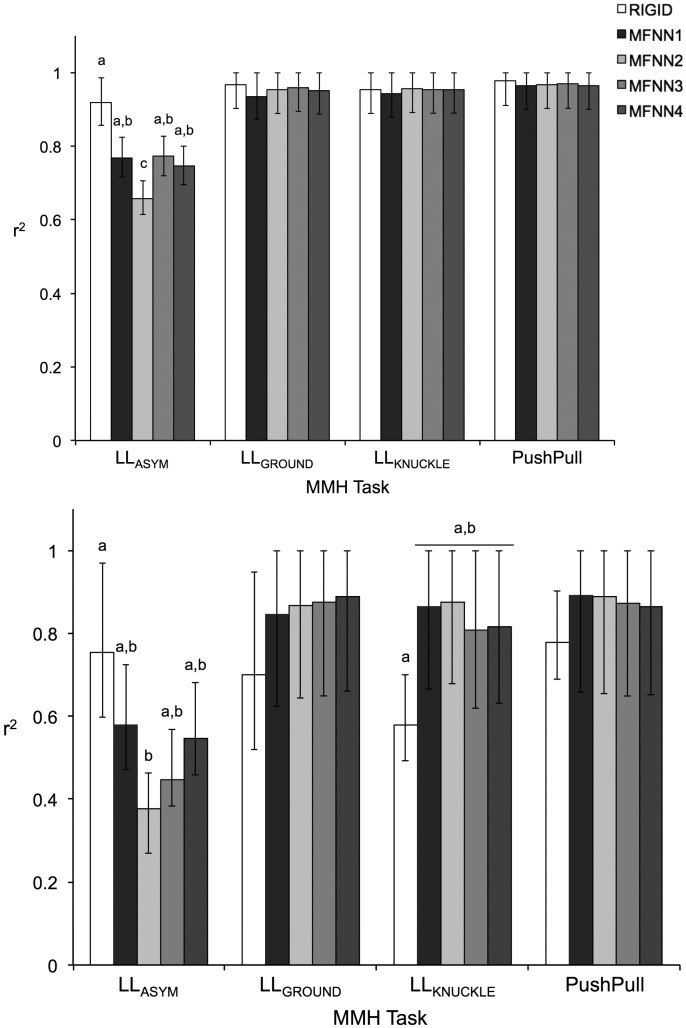
Coefficients of determination (r^2^) in the AP (**Top**) and the ML (**Bottom**) directions for each MMH task type with respect to the different methods for $T_{\text{Insole}}^{\text{Shoe}}$ . Pairs of values with different letters are significantly different within a given MMH task type, and error bars indicate 95% confidence intervals.

A summary of ANOVA results for all comparative measures is presented in Table 1. Only the main and interactive effects of *Task* and *Method* were significant. As shown in Figures 1, 2 and 3, comparative measures indicated relatively poorer performance during asymmetrical lifting/lowering *vs.* the other MMH tasks. In addition, for a given task the performance of a MFNN significantly varied depending on input variable sets, and MFNN3 generally outperformed the other MFNN models. Figures 2, 3 and 4 further highlight that the RIGID method produced comparable results to MFNN3 in some cases. Interestingly, the RIGID method (*vs*. MFNN methods) yielded significantly higher r^2^ values for asymmetrical lifting/lowering.

## 4. Discussion

This study examined two different approaches—rigid body transformation, and nonlinear mapping (MFNN)—to define a transformation between the coordinate systems of an IPM and the shoe itself (the latter being essentially equivalent to the global, or motion capture, coordinate system). Performance of the two approaches was assessed during several MMH activities. Overall, the results indicated that: (1) performance varied between MMH activity types regardless of the specific approach used; and (2) a MFNN performed better than or comparable to the rigid body transformation approach, depending on the specific input variable sets used.

The performance of the different transformation methods to define $T_{\text{Insole}}^{\text{Shoe}}$ varied with the MMH activity types. For example, asymmetric lifting resulted typically in larger errors and smaller r^2^ values than the other activities (Figures 2, 3 and 4). In the case of LL_GROUND_, LL_KNUCKLE_, and PushPull, mean RMSE values ranged from 6.5/2.3 mm to 16.6/6.8 mm in the AP/ML directions, depending on the transformation methods. Such errors are of similar magnitude to errors obtained during gait in earlier studies, which have reported mean RMSE values of 6.1/1.3 mm–13.7/7.3 mm in the AP/ML directions [10,17,18]. Chumanov *et al.* [17] and Fradet *et al.* [15] highlighted that relatively larger errors are obtained during the toe-off and/or the heel-strike phase of gait, and Fradet *et al.* further indicated that a potential error source is the deformation of shoes and insoles at these gait phases. The relatively poorer performance observed here for LL_ASYM_, therefore, could be explained by the fact that LL_ASYM_ requires more complex foot movements (e.g., turning, rolling, *etc.*) than the other activities. In addition, training for $T_{\text{Insole}}^{\text{Shoe}}$ was completed using only simple lifting activities (LL_GROUND_ and LL_KNUCKLE_), and which also likely contributed to a poorer performance for LL_ASYM_.

Use of a rigid body assumption to define the relationship between the insole and the shoe coordinate system may not be strictly valid, for at least two reasons. First, following the two-fold cross-validation, the rigid body transformation matrix was defined with one replication of LL_GROUND_ and LL_KNUCKLE_, and tested with the other replication of all MMH activities. Therefore, if the rigid body assumption is strictly valid, fairly good performance of $T_{\text{Insole}}^{\text{Shoe}}$ can be expected at least for LL_GROUND_ and LL_KNUCKLE_, even though the insole captures only compressive forces (while the force platform captures tri-axial forces) and the foot may move inside the shoe during the MMH activities. LL_GROUND_ and LL_KNUCKLE_, however, yielded RMSE and PAE values similar to PushPull (Figures 2,3), and smaller values of r^2^ in the ML direction than PushPull (Figure 4). In this study, all participants wore their own athletic shoes during data collection. An athletic shoe insole is pliant and soft, providing underfoot cushioning. Such a shoe insole could thus deform to varying degrees, depending on in-shoe foot loading patterns. This deformation may weaken the rigid body assumption for $T_{\text{Insole}}^{\text{Shoe}}$ . Second, two of the nonlinear mapping functions used here (*i.e*., MFNN3 and MFNN4) typically performed better than or comparable to the rigid body transformation matrix (though with some exceptions such as r^2^ values for LL_ASYM_). Recall that MFNN3 and MFNN4 used the ratios of active pressure sensors in the eight regions as part of the input variables, and information on such ratios likely reflected in-shoe foot loading patterns. It can thus be argued that MFNN3 and MFNN4 were trained to account, to some degree, for potential shoe insole deformation during the MMH activities.

Our study has some limitations of note. The MMH activities were performed in a laboratory environment, on a flat and regular surface. Irregularity of the ground surface will influence interactions between the shoe and the floor, and subsequently the degree of shoe deformation. Thus, future work is needed to assess IPM systems on under more general environmental conditions. In addition, the current sample size was somewhat small (*i.e.*, 10 participants). Though our results provided some direction for defining $T_{\text{Insole}}^{\text{Shoe}}$ , future work is also needed to consider more diverse populations (e.g., with obesity, foot deformities, *etc.*) for the rigid body transformation and nonlinear mapping approaches, so as to understand the generalizability of these approaches.

## 5. Conclusions/Outlook

Two different approaches—rigid body transformation, and nonlinear mapping—were examined to define a relationship between the insole and the shoe coordinate system, $T_{\text{Insole}}^{\text{Shoe}}$ , and the performance of the two approaches were examined during several MMH activities. MMH activity types affected the performance of the two approaches, with an asymmetric task (LL_ASYM_) yielding relatively poorer performance. The latter was attributed to potential shoe and insole deformations during LL_ASYM_. We further argued that the rigid body assumption to define a relationship between the insole and the shoe coordinate system may not be strictly valid, and suggested that a nonlinear mapping approach could account for potential shoe insole deformation during activities. Though our focus was MMH activities, a nonlinear mapping approach may also be effective in investigating postural balance during functional ambulation, since ambulatory activities such as stair climbing/descending likely induce shoe insole deformation. Existing studies using an IS (instrumented shoe) system [8,23] have reported mean RMSE errors or average absolute errors of <10 mm, and which is less than current errors since an IS system directly captures forces between the shoe (with externally attached tri-axial transducers) and the floor. Therefore, more efforts are needed to improve the performance of the methods to define $T_{\text{Insole}}^{\text{Shoe}}$ (e.g., incorporating foot kinematics along with IPM data, solving a transformation matrix, $T_{\text{Insole}}^{\text{Shoe}}$ without considering it homogeneous as in [20], *etc.*). In addition, and as noted in the limitations above, future work should examine the performance of methods to define $T_{\text{Insole}}^{\text{Shoe}}$ on different surfaces and/or with larger and more diverse populations.

## Figures and Tables

**Table 1.** Summary of ANOVA results (*F* value (*p*)) for the main and interaction effects of *Task, Side*, and *Method* on each comparative measure (significant effects are in bold).

**Measure**	**Task (T)**	**Side (S)**	**Method (M)**	**T × S**	**T × M**	**S × M**	**T × S × M**
	**F_(3,54)_**	**F_(1,18)_**	**F_(4,72)_**	**F_(3,54)_**	**F_(12,216)_**	**F_(4,72)_**	**F_(12,216)_**
RMSE_ML_	**232.06**	0.38	**20.61**	1.67	**4.00**	1.05	0.48
	**(<0.0001)**	(0.54)	**(<0.0001)**	(0.18)	**(<0.0001)**	(0.39)	(0.93)
RMSE_AP_	**72.55**	0.01	**10.97**	0.26	**8.34**	0.26	0.97
	**(<0.0001)**	(0.93)	**(<0.0001)**	(0.85)	**(<0.0001)**	(0.90)	(0.48)
PAE_ML_	**153.77**	1.35	**9.74**	2.61	**2.33**	1.60	0.34
	**(<0.0001)**	(0.26)	**(<0.0001)**	(0.06)	**(0.0081)**	(0.19)	(0.98)
PAE_AP_	**56.77**	0.00	**6.85**	0.49	**5.03**	0.15	1.41
	**(<0.0001)**	(0.99)	**(<0.0001)**	(0.69)	**(<0.0001)**	(0.96)	(0.16)
r^2^_ML_	**14.53**	0.15	**3.61**	0.79	**5.65**	0.21	0.82
	**(<0.0001)**	(0.71)	**(0.0097)**	(0.51)	**(<0.0001)**	(0.93)	(0.63)
r^2^_AP_	**25.38**	0.43	**4.00**	0.29	**3.55**	0.81	0.97
	**(<0.0001)**	(0.52)	**(0.0056)**	(0.83)	**(<0.0001)**	(0.52)	(0.48)
